# The association between micronutrient powder delivery patterns and caregiver feeding behaviors in rural China

**DOI:** 10.1186/s12889-022-13726-4

**Published:** 2022-07-16

**Authors:** Rong Liu, Ruixue Ye, Qingzhi Wang, Lucy Pappas, Sarah-Eve Dill, Scott Rozelle, Huan Zhou

**Affiliations:** 1 Institute for Disaster Management and Reconstruction, Sichuan University, Huanghe Middle Road Section 1, Chengdu, 610041 China; 2 Department of Health Behavior and Social Medicine, West China School of Public Health and West China Fourth Hospital, Sichuan University, No.16 South Renmin Road 3 Section, Chengdu, 610041 China; 3 Stanford Center on China’s Economy and Institutions, Freeman Spogli Institute for International Studies, Stanford University, Stanford, CA 94305 USA

**Keywords:** Micronutrient powders, Adherence, Proper usage, Feeding behavior, Delivery patterns, China, Rural

## Abstract

**Background:**

High adherence and proper usage of micronutrient powder (MNP) influence child nutritional outcomes, yet few studies explore the role of delivery patterns. This study explores the association between MNP delivery patterns and MNP feeding behaviors among Han and minority caregivers in rural Western China.

**Methods:**

In August 2019, a total of 1021 caregiver-child pairs were selected through a four-stage cluster sampling process. A cross-sectional survey collected information on caregiver demographics, MNP delivery patterns (channel and frequency), and MNP feeding behaviors (proper usage and adherence). Using logistic regression, we examined which delivery channels and delivery frequencies were associated with proper usage and high adherence.

**Results:**

The results indicated that minority caregivers had lower levels of proper MNP usage than did Han caregivers (89.2%), with Tibetan caregivers’ reporting the lowest rates of adherence (32.6%). Logistic regression revealed that that township-based channel was significantly correlated with proper usage among Tibetan and Yi caregivers (Odds Ratio, OR = 2.0, *p* < 0.01; and OR = 3.5, *p* < 0.001). Overall, the township-based and home-visit channels were significantly correlated with high adherence (OR = 1.7 and OR = 2.3, respectively; *p* < 0.001); delivery frequency was significantly correlated with high adherence (2 months: OR = 2.2, *p* < 0.001 and ≤ 1 month: OR = 3.5, *p* < 0.001) but not correlated with proper usage among the whole sample and individual ethnic groups.

**Conclusions:**

In conclusion, the study finds evidence of a correlation between MNP delivery channel and both proper usage and high adherence as well as a correlation between MNP delivery frequency and high adherence.

**Supplementary Information:**

The online version contains supplementary material available at 10.1186/s12889-022-13726-4.

## Background

Child undernutrition is a serious global health problem that causes adverse outcomes in short- and long-term development. “Undernutrition” is a deficiency in the intake of energy and/or nutrients by children, resulting in four forms: wasting (low weight-for-height), stunting (low height-for-age), being underweight (low weight-for-age), and micronutrient deficiencies (deficiencies in vitamins and minerals) [1]. All four forms of undernutrition jeopardize the health, growth, development, and survival of children, and all can cause negative, irreversible effects [2]. Specifically, children who suffer from undernutrition can experience significant delays in their cognitive and psychomotor development as well as weakened resistance and immunity to diseases and increased rates of child morbidity and mortality [3]. Undernutrition has been shown to lead to lifelong consequences that involve adverse health outcomes, including worsened intellectual and reproductive abilities as well as increased risk of hypertension, diabetes, and psychiatric disorders in adults [2, 4, 5]. These adverse health outcomes may affect human capital and economic productivity, making undernutrition more than just a public health concern [6, 7].

The literature has demonstrated that undernutrition is a particularly serious problem in many low- and middle-income countries (LMICs) [8–10]. In LMICs, the prevalence of overall childhood stunting among children under the age of 5 years ranges from 21.5 to 32.4%, and the rates of similarly-aged children being underweight ranges from 27.3 to 27.6% [8]. Although less widespread than stunting and being underweight, childhood wasting affects 4.9 to 7.9% of children in LMICs [8]. The prevalence of micronutrient deficiencies in children ranges from 5 to 38.8% [11–13]. In addition, an estimated 29% of children in LMICs have vitamin A deficiencies [14], and more than 50% of children in LMICs suffer from zinc deficiencies [15].

Fortunately, programs that distribute micronutrient powder (MNP) to households with infants and young children have the potential to reduce widespread undernutrition; however, research in LMICs frequently finds evidence of inconsistent implementation among programs as well as variations in adherence to the MNP programs [16, 17]. Internationally, MNP programs have been implemented to address child undernutrition, and studies have found that such programs, when implemented fully, can lead to significant declines in undernutrition, as is the case in Asia, Africa, and the Caribbean [18–20]. Previous research also suggests that the delivery channels of MNP (how MNP is distributed to caregivers) affects MNP coverage and caregiver adherence to MNP. One study in Nepal, which compared different MNP delivery channels (e.g., distribution of MNP by community health volunteers versus at health facilities), reported that both channels led to significant but incomplete (by themselves) coverage of MNP and concluded that multiple delivery patterns were needed for successful MNP program implementation [21]. A study in Uganda reported that delivery pattern and program adherence are correlated and that a community-based delivery channel resulted in higher levels of MNP adherence than did a health facility-based delivery channel (58.3% compared to 31.4%) [22].

Similar to research in other LMICs, several studies have found high rates of child undernutrition in rural China [23, 24]. In China, a middle-income country, there is a large and substantial number of undernourished children who live in rural areas. The National Institute of Nutrition and Health reported that approximately 7 million children (under the age of 5) in rural China are stunted (20.3%), and 2 million are underweight (8.0%) [25, 26]. As recently as 2019, the prevalence of iron-deficiency anemia (IDA) was reported to be around 50% in Western and Southern rural China, which is twice the overall prevalence of IDA across all of China’s rural areas (25.1%) [27]. When looking closely at those affected by undernutrition in rural China, research finds that rates of undernutrition are higher among minority ethnic groups (such as the Tibetan and Yi areas) than China’s majority ethnic group (Han) [28, 29].

To address this health issue, China’s public health system has implemented MNP programs across the country, following recommendations from the World Health Organization (WHO) [30]. In 2012, the National Health Commission, in cooperation with the All-China Women’s Federation, implemented a nutrition improvement project for children in poverty-stricken areas in 21 provinces [30]. The program, titled Child Nutrition Improvement Program, provides free MNP (*yingyangbao* in Mandarin) in the form of a soy-based powder with added micronutrients, such as iron, zinc, and vitamins, to families with children aged 6 to 24 months across rural China [30, 31].

Although the implementation of China’s MNP program was supposed to be carried out uniformly across the nation, there is evidence that adherence to MNP in rural Western China varies across ethnic groups. Specifically, the findings indicate that Han caregivers typically have higher adherence to MNP than do minority groups, such as the Yi and Tibetan [32–34]. In rural Western China, ethnic groups have distinct food cultures and feeding practices, in addition to different lifestyles [24, 28, 35], that may influence how caregivers access MNP and how they feed MNP to their children. To the best of our knowledge, no study has examined the association between MNP implementation success/failure and the delivery patterns and feeding behaviors of MNP across different ethnic groups in China.

Given the gap in the literature, this paper has two main objectives. First, we investigate the differences in MNP delivery patterns (delivery channels and frequencies of delivery) and caregiver feeding behaviors of MNP (proper usage and adherence) among Han, Tibetan, and Yi caregivers. Second, we explore the associations between delivery patterns (delivery channels and frequencies of delivery) and feeding behaviors (proper usage and adherence) of MNP among these three ethnic groups.

## Methods

### Sampling

In August 2019, the research team conducted a cross-sectional study in rural areas of Sichuan Province, located in Western China. Sichuan Province is home to many ethnic minority groups, there is the largest inhabited area of the Yi ethnic group, the second-largest inhabited area of the Tibetan ethnic group [36]. Within the total population of Sichuan Province (83.67 million), 93.2% are ethnically Han (the majority in China), and the remaining 6.7% include non-Han ethnic minority populations, including Tibetan (1.7%) and Yi (3.1%), other minorities including Qiang, Miao, Hui, Mongolian et.al total accounting for 2% [36]. To capture the ethnic diversity of Sichuan, our sample comprised children and caregivers from Han, Tibetan, and Yi households.

**Fig. 1:**
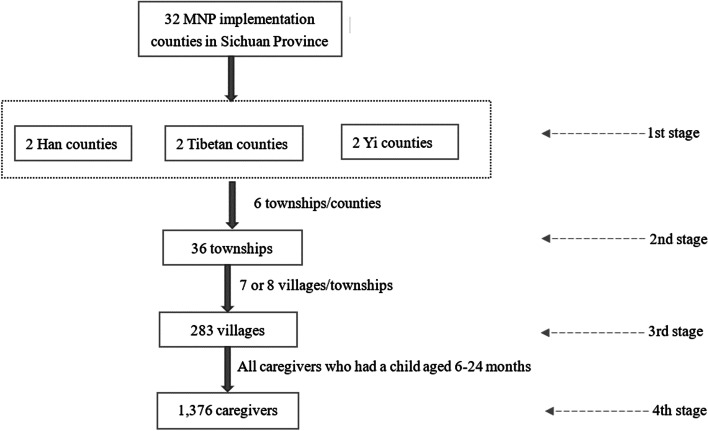
The sampling frame for the survey of caregivers and their children (ages 6–24 months) in rural Sichuan Province, China

We used a four-stage cluster sampling method to select the sample (Fig. 1). First, we obtained a list of 32 known MNP program implementation sites in Sichuan province (where MNP was distributed free of charge by the government) from the Sichuan Provincial Maternal and Child Health Care Hospital (*Sichuan sheng fu you bao jian yuan*). From the 32 counties, a total of six, two Han and four minority counties (which included two Tibetan counties and two Yi counties), were randomly selected. Counties were determined to be minority counties if the majority of the county population identified as one non-Han ethnic minority or two or more minority ethnicities (Tibetan or Yi) [37]. In this sample, Tibetan populations account for 92 and 72.47% of the two Tibetan counties [38, 39], while Yi populations account for 97.5 and 97.1% of the two Yi counties [40, 41]. The Han counties were determined when the majority of the county identified as Han. In the Han counties, Han populations accounted for 99.6 and 99.8% of the total of the two county populations [42, 43].

Second, six townships within each of the six selected counties were randomly selected, totaling 36 townships. Third, from each of the 36 townships, the research team randomly selected six villages. If a village had a population of fewer than 800 people, we combined two neighboring villages (each with fewer than 800 people) and considered them as one village-level sampling unit. In total, 283 villages were selected. Last, our team obtained a list of all registered births over the previous 24 months (August 2017– February 2019) from local officials in each village. This list was used to confirm target child age range (6–24 months) during the survey period by calculating the difference between the survey date and the registered birth date.

In this study, ‘caregiver’ refers to the person in a family who is primarily responsible for taking care the child on a daily basis (the primary caregiver). Since China’s MNP program provides free MNP for children aged 6–24 months, all children in this target age range with their primary caregiver were deemed eligible to be enrolled in the survey. Finally, in the 283 villages, a total of 1376 (Fig. 1) pairs were eligible for the survey. However, 140 failed to enroll in the study due to various reasons: (A) the caregiver and the child had migrated to another city or province to live or work; (B) the caregiver was absent and would not return to the residence during the time of the survey; (C) the caregiver’s child was sick, and the caregiver was unable to be interviewed; or (D) the caregiver refused to be interviewed. Of the 1236 caregivers who enrolled in the survey, 1021 were included in the final analytical sample. A total of 215 caregivers (exclusion rate 17.39%) were not included in the final analysis due to various reasons: (a) their children died before the survey began; (b) they had never heard of or seen MNP; (c) the caregiver left during the questionnaire administration due to personal reasons and failed to return; (d) the caregiver initially accepted the investigation but refused to complete the questionnaire due to fatigue or lack of interest; or (e) the caregiver or investigator omitted an item on the survey. After running an attrition analysis, our results mainly showed no significant differences between the 1021 caregivers who were included and the 215 caregivers who were not included in the final analytical sample (see the Appendix Table 1 in the Additional File 1). The only significant differences between included and excluded caregivers were educational background (*p* = 0.002) and delivery frequency (*p* = 0.001).

### Data collection

Data for the study were collected by trained enumerators using a structured survey questionnaire. The questionnaire was developed by the research team after a comprehensive literature review and two rounds of Delphi expert consultation. The research team piloted the survey in two non-sample villages with 20 caregivers. The questionnaire was then revised to form the final survey.

Accompanied by a local county doctor, trained enumerators visited households and were introduced to each household’s primary caregiver. To overcome the language barriers in ethnic minority communities, we recruited and trained local volunteers to help translate the Mandarin survey questionnaire into proper dialects. In total, the survey gathered three blocks of data: (a) caregiver MNP feeding behaviors with a focus on the provision of MNP to children; (b) channels of access to MNP (i.e., how the family came into possession of MNP); and (c) sample demographic characteristics (available in Appendix Table 2 in Additional File 1).

The first block of data included information on caregiver MNP feeding behaviors. First, caregivers were asked to report their usage of MNP, including how they usually fed MNP to their children, by choosing one of three answers: (a) 1 = adding MNP to warm boiling water and stirring into a paste, (b) 2 = mixing MNP with other supplementary food, or (c) 3 = other. According to the official MNP feeding instructions from the National Health Commission [2], “proper usage” is defined as “adding MNP to warm, boiling water and stirring into a paste, or as mixing MNP with other supplementary food” (Answers 1 or 2). Any other MNP usage methods not described by the official instructions was determined “not proper usage.” Second, caregivers were asked about their adherence to the frequency of provision of MNP to their children and reported the number of MNP sachets that they fed their child every week, by choosing either 1 = 4 sachets or more/week or 0 = fewer than 4 sachets/week. “high adherence” was defined as feeding a child at least four MNP sachets every week, and “low adherence” was defined as feeding a child fewer than four MNP sachets every week. These definitions of adherence were in accordance with the official government MNP instructions and previous studies [10, 18, 44, 45].

The second block of data collected was on MNP delivery patterns. First, we asked caregivers to choose which type of delivery channel they used to access MNP: (a) 1 = village-based channel (village health office, village activity room, or village committee location); (b) 2 = township-based channel (township health center); or (c) 3 = home-visit delivery channel (caregiver’s household by a home visit from the village doctor or village women’s director). Second, we asked caregivers how often they accessed MNP: (a) 1 = every three or more months (≥3 months); (b) 2 = every two months (2 months); or (c) 3 = every month or multiple times every month (≤1 month).

The third block of data collected was demographic characteristics of children, caregivers, and households. For child characteristics, caregivers reported their child’s gender, age in months, and health status. Regarding health status, caregivers were asked to report whether their child’s health was (a) 1 = very poor, (b) 2 = poor, (c) 3 = fair, (d) 4 = good, or (e) 5 = very good. For caregiver characteristics, caregivers reported their gender, age in years, level of educational attainment (never attended school; did not complete elementary school; completed elementary school; completed primary school; completed high school or above), ethnicity (Han, Tibetan, or Yi); and occupation (farmers, full-time stay-at-home parents, or other). Finally, we collected demographic information on household characteristics, which included annual household income (<RMB 1.2 k, ~RMB 1.2 k, ~RMB 2.5 k, ≥RMB 5 k).

### Statistical analysis

The statistical analysis consists of three parts. First, we use descriptive analyses (means, standard deviations or SD, and shares) to present demographic characteristics of children and their caregivers. Second, we report the prevalence of proper usage and adherence of MNP, using univariate analysis (χ^2^ test) to explore the association of MNP feeding behaviors (proper usage and adherence) with MNP delivery patterns (delivery channels and frequency) among Han, Tibetan, and Yi groups. Third, we use logistic regression to examine which delivery channels and delivery frequencies are associated with proper usage and high adherence. In this study, we take the village-based delivery channel and the lowest frequency (≥3 months) as reference. Odds ratios for the unadjusted (without the controlled variables) and adjusted results are reported; *p*-values below 0.05 are considered statistically significant. All analyses were carried out using Stata 14 (Stata Corp, 2015).

### Ethics statement

This study has been performed in accordance with the Declaration of Helsinki and has been received ethical approval from the Sichuan University Medical Ethical Review Board (Approval No. K2018103). Before conducting interviews, trained enumerators explained the study aims, process, potential risks and benefits, privacy measures that would be taken, rights and duties of the individual, and contacts for the study to participating caregivers. Enumerators also presented each caregiver with a standardized document that contained the above information. Participating caregivers signed the consent forms for their own and their child’s involvement in the program if applicable, other participating caregivers also gave their oral consent.

## Results

### Demographic characteristics

The demographic characteristics of the total sample, Han subsample, Tibetan subsample, and Yi subsample are reported in Table 1. A total of 1021 pairs of caregivers and children were included in our study, including 352 Han, 307 Tibetan, and 362 Yi caregiver-child pairs. Less than half of the sample children (48.1%) were female, and the mean child age was 18.9 months (Standard Deviation, SD = 5.8 months). The mean child health status score was 4.4 (SD = 0.8), which was between 4 = good health and 5 = very good health. There were no significant differences in the characteristics of the sample children among the three ethnic groups.

**Table 1:** Demographic characteristics of sample child and caregiver pairs from rural western China (*N* = 1021)

Variable	Total (*N* = 1021)		Han (*n* = 352)		Tibetan (*n* = 307)		Yi (*n* = 362)		*p*-value
	*N* / Mean	(*%*) / (SD)	*n* / Mean	(*%*) / (SD)	*n* / Mean	(*%*) / (SD)	*n* / Mean	(*%*) / (SD)	
Child characteristics									
Gender									0.941
Female	491	(48.1%)	169	(48.0%)	150	(48.9%)	172	(47.5%)	
Male	530	(51.9%)	183	(52.0%)	157	(51.1%)	190	(52.5%)	
Age (months)	18.9	(5.8)	19.36	(5.6)	18.59	(5.9)	18.72	(5.9)	0.474
Health status	4.4	(0.8)	4.18	(0.7)	4.49	(0.8)	4.54	(0.7)	0.156
Caregiver characteristics									
Gender									0.059
Female	912	(89.3%)	324	(92.0%)	265	(86.3%)	323	(89.2%)	
Male	109	(10.7%)	28	(8.0%)	42	(13.70%)	39	(10.8%)	
Age (years)	35.9	(12.9)	39.3	(13.5)	35.9	(12.9)	32.6	(11.3)	0.003
Educational background									< 0.001
Never went to school	476	(46.6%)	40	(11.4%)	153	(49.8%)	283	(78.2%)	
Did not complete elementary school	117	(11.5%)	54	(15.3%)	36	(11.7%)	27	(7.5%)	
Completed elementary school	132	(12.9%)	51	(14.5%)	46	(15.0%)	35	(9.7%)	
Completed primary school	170	(16.7%)	129	(36.7%)	31	(10.1%)	10	(2.8%)	
Completed high school or above	126	(12.3%)	78	(22.2%)	41	(13.4%)	7	(1.9%)	
Occupation									< 0.001
Farmer	418	(40.9%)	45	(12.8%)	84	(27.4%)	289	(79.9%)	
Full-time stay-at-home parent	466	(45.6%)	256	(72.7%)	157	(51.1%)	53	(14.6%)	
Other	137	(13.5%)	51	(14.5%)	66	(21.5%)	20	(5.5%)	
Annual household income (AHI)									< 0.001
< RMB 1.2 k	326	(31.9%)	14	(3.9%)	48	(15.6%)	264	(72.9%)	
~ RMB 1.2 k	305	(29.9%)	84	(23.9%)	143	(46.6%)	78	(21.6%)	
~ RMB 3.5 k	146	(14.3%)	70	(19.9%)	64	(20.9%)	12	(3.3%)	
≥ RMB 5 k	244	(23.9%)	184	(52.3%)	52	(16.9%)	8	(2.2%)	

Notes: Child age, child health status, and caregiver age are listed in mean (SD); all other variables are listed in frequency (percentage). Child health status was reported by caregivers on a scale of 1 to 5: 1 = very poor; 2 = poor; 3 = fair; 4 = good; or 5 = very good

Regarding caregiver characteristics, the majority of caregivers were female (89.3%), and the mean age of the sample caregivers was 36.9 years. There were significant differences in caregiver educational attainment and occupation among the ethnic groups (*p* < 0.001). In the case of educational attainment, 49.8% of Tibetan and 46.6% of Yi caregivers reported that they had never attended any level of school, while Han caregivers had significantly higher levels of educational attainment (*p* < 0.001). The majority of Yi caregivers were farmers (79.8%), while 14.6% were full-time stay-at-home parents, and 5.5% were other (such as individually self-employed drivers, for example). In contrast, the majority of Han and Tibetan caregivers were stay-at-home parents (72.7 and 51.1%, respectively).

According to the data on household characteristics, 31.9% of caregivers had annual household incomes (AHI) in the lowest income bracket (<RMB 1.2 k) and 23.9%, in the highest bracket (≥RMB 5 k). When comparing AHI among different ethnic groups, we find that Han households reported significantly higher AHI than did Tibetan and Yi households (*p* < 0.001). Specifically, more than half of Han households (52.3%) reported AHI in the highest income category and only 3.9% of households, in the lowest income level. For the average AHI of Tibetan households, 15.6% were in the lowest AHI level, and 16.9% were in the highest level. Finally, the majority of Yi households (72.9%) reported incomes in the lowest AHI level, while only 2.2% of Yi households reported incomes in the highest AHI level.

### Distribution of delivery patterns and feeding behaviors across ethnic groups

Table 2 shows the results on MNP delivery patterns and MNP feeding behaviors among the caregivers in the full sample and in the ethnic subsamples. Across the full sample, the most common delivery pattern was the township-based channel (53.1%), followed by village-based (28.5%) and home visit-based (18.4%). The most prevalent delivery frequency was every month or multiple times per month (49.9%), followed by every 3 or more months (39.1%). Overall, 74.0% of all caregivers reported proper usage, and 75.1% reported high adherence to MNP feeding guidelines.

**Table 2:** Differences in MNP delivery patterns and MNP feeding behaviors between ethnic groups

Item	Total (*N*)	(%)	Han (*n*)	(%)	Tibetan (*n*)	(%)	Yi (*n*)	(%)	*p*-value
Delivery pattern									
Channel^a^									< 0.001
Village-based	291	(28.5%)	45	(12.8%)	60	(19.5%)	186	(51.4%)	
Township-based	542	(53.1%)	289	(82.1%)	184	(59.9%)	69	(19.1%)	
Home visit-based	188	(18.4%)	18	(5.1%)	63	(20.5%)	107	(29.6%)	
Frequency									< 0.001
≥ 3 months	399	(39.1%)	127	(36.1%)	95	(30.9%)	177	(48.9%)	
2 months	113	(11.1%)	54	(15.3%)	28	(9.1%)	31	(8.6%)	
≤ 1 month	509	(49.9%)	171	(48.6%)	184	(59.9%)	154	(42.5%)	
Feeding behavior									
Proper usage^b^									
Yes	755	(74.0%)	314	(89.2%)	224	(73.0%)	217	(59.9%)	
No	266	(26.1%)	38	(10.8%)	83	(27.0%)	145	(40.1%)	< 0.001
Adherence^c^									
Low	254	(24.9%)	77	(21.9%)	100	(32.6%)	77	(21.3%)	
High	767	(75.1%)	275	(78.1%)	207	(67.4%)	285	(78.7%)	0.001
Total	1021	(100.0%)	352	(34.5%)	307	(30.1%)	362	(35.5%)	

Notes: MNP refers to micronutrient powder

^a^ Village-based delivery channel: MNP was distributed at the village health office, village activity room, or village committee location; township-based: MNP was distributed at the township health center; home visit-based: MNP was distributed to the household in a home visit

^b^ Proper usage (Yes) refers to following the MNP feeding instructions: Adding MNP to warm boiling water and stirring into paste; or mixing MNP with other supplementary food. Proper usage (No) refers to other usage

^c^ High adherence: ≥4 MNP sachets were consumed every week, low adherence: < 4 MNP sachets were consumed every week

Table 2 also shows significant differences in MNP delivery patterns and MNP feeding behaviors between Han, Tibetan, and Yi caregivers. There were significant differences in the most common delivery channel among the ethnic groups. Whereas most Han and Tibetan caregivers accessed MNP through the township-based channel (82.1 and 59.9%, respectively), most Yi caregivers accessed MNP through the village-based channel (51.4%) (*p* < 0.001). There also were significant differences in MNP access frequency among the families in the different ethnic groups. Han and Tibetan caregivers accessed MNP at a significantly higher frequency (48.6 and 59.9% accessed MNP ≤ 1 month, respectively) than did Yi caregivers (48.9% accessed MNP ≥ 3 months; *p* < 0.001).

The results also show that there were significant differences in MNP feeding behaviors among Han, Tibetan, and Yi caregivers. Han caregivers had significantly higher rates of proper usage (89.2%) than did Tibetan (73.0%) and Yi (59.9%) caregivers (*p* < 0.001). In addition, Tibetan caregivers had significantly higher rates of adherence to MNP (32.6%) than did Han (21.9%) and Yi (21.3%) caregivers (*p* = 0.001).

### Associations between MNP delivery patterns and feeding behaviors across ethnic groups

Table 3 presents the results of the multivariate analysis of MNP delivery patterns (channel and frequency) and MNP feeding behaviors (proper usage and adherence to MNP guidelines by caregivers) among the sample’s ethnic groups. The univariate analysis results (unadjusted logistic regressions) and overall results of the multivariate analysis (when the regression analysis controlled for demographic characteristics) are displayed in Appendix Table 3 and Appendix Table 4, respectively, in Additional File 1.

**Table 3:** Multivariate analysis of proper usage and adherence to MNP among different ethnic groups using adjusted logistic regression (after control variables)

Variable	Proper usage^b^ (OR)				Adherence^c^ (OR)			
	Total	Han	Tibetan	Yi	Total	Han	Tibetan	Yi
Channel^a^ (Village-based as reference)								
Township-based	2.6***	2.1	2.0**	3.5***	1.7***	2.2**	1.4	2.6***
	(0.5)	(0.9)	(0.6)	(1.2)	(0.3)	(0.8)	(0.5)	(0.9)
Home visit-based	1.1	0.9	0.9	1.8	2.3***	N/A	1.5	4.8***
	(0.2)	(0.7)	(0.3)	(0.8)	(0.6)	N/A	(0.5)	(2.4)
Frequency (≥3 months as reference)								
2 months	0.8	1.1	0.5	1.8	2.2***	2.6**	1.2	3.9***
	(0.2)	(0.5)	(0.2)	(0.9)	(0.6)	(1.1)	(0.6)	(1.9)
≤ 1 month	0.9	1.6	0.8	1.0	3.5***	3.1***	4.3***	5.8***
	(0.2)	(0.6)	(0.2)	(0.3)	(0.6)	(0.9)	(1.5)	(1.8)

Notes: MNP refers to micronutrient powder; odds ratios were reported; standard errors in parentheses. The results of control variables are not reported here. Adjusted logistic regression (after control variables) used the following for reference: High adherence = 1, Low adherence = 0. Proper usage = 1, Improper usage = 0

^**a**^ Village-based delivery channel: MNP was distributed at the village health office, village activity room, or village committee location; township-based: MNP was distributed at the township health center; home visit-based: MNP was distributed to the household in a home visit

^b^ Proper usage (Yes) refers to following the MNP feeding instructions: Adding MNP to warm boiling water and stirring into paste; or mixing MNP with other supplementary food. Proper usage (No) refers to other usage

^**c**^ High adherence: ≥4 MNP sachets were consumed every week, low adherence: < 4 MNP sachets were consumed every week

*p < .05. **p < .01. ***p < .001

Compared to the village-based channel, the township-based channel was significantly correlated with proper usage of MNP among the full sample (Odds Ratio, OR = 2.6, *p* < 0.001). An examination of the results within individual ethnic groups shows that the township-based channel was significantly correlated with proper usage of MNP among Tibetan and Yi caregivers (OR = 2.0, *p* < 0.01, OR = 3.5, respectively; *p* < 0.001). Delivery frequency was not significantly correlated with proper usage of MNP for the whole sample or among the ethnic subsamples.

Regarding adherence to MNP, overall, the township-based and home-visit channels were significantly correlated with high adherence (OR = 1.7 and OR = 2.3, respectively; *p* < 0.001). Among Han caregivers, the township-based channel was significantly correlated with high adherence (OR = 2.1, *p* = 0.9). The township-based and home-visit channels were significantly correlated with high adherence for Yi caregivers (OR = 2.6 and OR = 4.8, respectively; *p* < 0.001). Further, higher delivery frequency was significantly correlated with high adherence across the whole sample (2 months: OR = 2.2, *p* < 0.001; ≤1 month: OR = 3.5, *p* < 0.001). The results also show that, among each ethnic group, accessing MNP each month or multiple times per month was significantly correlated with high adherence (OR = 5.8, OR = 4.3, and OR = 3.1, respectively; *p* < 0.001), while accessing MNP every 2 months was significantly correlated with high adherence for Han and Yi caregivers (OR = 2.6 and OR = 3.9, respectively; *p* < 0.001).

## Discussion

This is the first study to explore the associations between MNP delivery patterns (delivery channel and frequency) and caregiver MNP feeding behaviors in the context of an MNP implementation program in rural China. Our results indicate that, across three ethnic groups, each group accesses MNP through different channels and at different frequencies. The data show that Han caregivers have the highest levels of proper MNP usage, whereas Yi and Han caregivers have the highest rates of adherence to the prescribed MNP program. Finally, our results show that delivery patterns influence proper MNP usage and high adherence to MNP. Specifically, we find that a township-based delivery channel leads to proper usage of MNP among Tibetan and Yi caregivers, whereas accessing MNP at a higher frequency correlates to higher adherence among Han, Tibetan, and Yi caregivers.

Generally, the attrition analysis combined with the estimated proportion of samples excluded to total samples were served as the most effective measurements [46]. Previous study found higher education level usually correlates to higher non-response rate [47, 48]. But as for this study, our samples have relatively low education level since it focused in rural China, which may impact the differences of educational level and also delivery frequency. Despite identifying such differences, the exclusion rate (17.39%) is considered acceptable given the fact that the excluded participants did not meet our study objectives or were missing crucial variables for inclusion [49]. In summation, we feel confident in our included samples representation regarding the most other variables included in this study shows no difference. Another point worth mentioning is that most Han and Tibetan primary caregivers are full-time stay-at-home parents, which is in line with households throughout rural China. In our sample, the majority of primary caregivers are female (most likely mothers or grandmothers), which is another similarity to other samples in rural China. Previous studies in rural China have demonstrated high quantities of left-behind children and female caregivers, who are financially supported by caregivers who migrate away from home to find work outside the household [50–52]. Thus, many female primary caregivers in rural China are solely responsible for looking after children and managing household affairs [53], while other household members work outside the home to financially support the family [54, 55].

The results show that caregivers of different ethnicities access MNP through different delivery patterns. Although few studies have compared differences in MNP delivery channels between Chinese ethnic groups, our finding is consistent with studies that find that delivery patterns differ among different groups in different settings [21, 56, 57]. In Vietnam, Nguyen et al. (2016) found that caregivers from ethnic minorities need a complementary delivery model, beyond a health services delivery channel, to properly use MNP, due to their being geographically marginalized, with limited access to the health system [57]. As for our sample, the most common way that Tibetan and Han caregivers access MNP is through a township-based channel, and they access MNP at the highest frequency: every month or multiple times each month. Through the township-based channel, MNP is distributed to caregivers at local township health centers. Regular gatherings at township markets, called “Ganchang” in Mandarin, are a cultural tradition for many residents of rural China [58]. In addition to attending markets for trading, socializing, and viewing entertainment, rural residents can more frequently visit their local township health centers to obtain MNP. Notably, very few Yi caregivers in our sample access MNP through the township-based channel. Instead, Yi caregivers use the village-based channel and access MNP every three or more months (the lowest measured frequency). Another possible influence on delivery pattern differences is the local topography where ethnic groups live. In this study, the terrain of the sample area in the Tibetan community is dominated by plateaus, while the Han and Yi areas are mountainous regions. Local topographical features, coupled with the human resources available to MNP health programs, may dictate how MNP distributors adopt specific distribution channels and frequencies to reach certain populations and groups [36].

In addition to differences in MNP delivery patterns among ethnic groups, there are distinct differences in caregiver MNP feeding behaviors. In China, different ethnic groups have different feeding and food cultures, which may influence how they feed MNP to children [35]. For example, Han caregivers, on average, report a higher prevalence of proper MNP usage than do caregivers of children in the minority samples, which may be due to their feeding cultures and acceptance of nationwide health programs, such as this MNP implementation program. Another possible reason for differences among MNP feeding behaviors may be an issue of informational access. Among our sample, there is a high proportion of Yi and Tibetan caregivers who never attended school, which makes them more likely to be unable to read MNP written instructions and, thus, less likely to display correct feeding behaviors. Conversely, Han caregivers in our sample reported higher levels of education (and were nearly all able to read Chinese characters), which may have influenced their ability to understand the written MNP feeding instructions and other public information regarding MNP [44, 59]. Beyond educational attainment, language barriers may prohibit ethnic minority caregivers from correctly interpreting MNP usage instructions. Distinct from Mandarin, Tibetan and Yi languages have their own characters and spellings, which makes MNP instructions written in Mandarin largely indecipherable [60]. To address these language barriers, a wider range of public information, such as pamphlets, brochures, and nutrition packaging and instructions, in more dialects and languages is needed to more effectively distribute MNP. Further, MNP distributors should disseminate information about the usage of MNP to mothers and other caregivers in person, as well as through local village broadcasts that can be projected over loudspeakers in village centers. One study conducted in Vietnam found that training MNP distributors on how to better communicate with caregivers led to better program implementation [57]. Another study, in Peru, found that MNP distributors could influence caregivers’ feeding behaviors (acceptance and usage of MNP) by how they presented MNP to caregivers [61]. These findings are in line with studies in Nigeria, Kenya, Ethiopia and Ghana, which confirm that better communication and support from distributors positively influence MNP adherence [56, 62–65].

We also find differences in levels of adherence to MNP between ethnic groups. Yi and Han caregivers report the highest rates of adherence to MNP. Tibetan caregivers in our sample are the only group who falls below the recommended adherence rate by the National Health Commission (70.0%) [45]. Although this is a public health concern, when comparing these results from a recent meta-analysis on MNP adherence in LMICs, we find that the average adherence rate of our sample is slightly higher than a pooled adherence rate of 63.28% [44] and is similar to the rates reported in other studies in LMICs (65–81%) [66–70]. Moreover, our samples’ adherence rates were higher than those in Cambodia (56%) [71] and Mali (65%) [66] but slightly lower than the adherence rates in Mongolia and India (88 and 84%, respectively) [72].

Previous research has neglected to evaluate the factors of proper MNP usage [10]. Our results, however, provide preliminary evidence that the type of delivery channel, not the frequency of the delivery, influences proper usage. Our results show that the township-based channel leads to proper MNP usage for caregivers, overall, as well as for Yi and Tibetan caregivers, individually. This finding indicates that township doctors may be playing an important role in distributing MNP to minority caregivers. Previous studies have identified townships doctors as an integral health communication channel for providing MNP-related information to caregivers in rural China, which was critical to caregivers knowledge of MNP and then led to better feeding behavior [73]. Specifically, township doctors provide key MNP information that is critical to educating caregivers on better MNP feeding behaviors [74] . For these reasons, township doctors could be promoted as more important actors in future policy or interventions that aim to improve MNP usage among minority populations.

Regarding adherence, we found that the township-based and home-visit channels are significantly correlated with high adherence to MNP, regardless of ethnicity. Our findings are in line with those of studies from LMICs (Nepal, Uganda, and Vietnam) that demonstrate that delivery patterns are related to MNP adherence [21, 22, 57]. The study conducted in Nepal concluded that multiple delivery patterns may be necessary for successful MNP program implementation [21], while the study in Vietnam found that delivery through local community health centers was a key factor to high MNP adherence [57]. Other international evidence suggests that community-based distribution channels not only result in higher coverage but also influence caregivers’ MNP adherence rates [67, 72]. These studies confirm that delivery patterns matter for MNP program implementation and that specific delivery patterns should be adopted based on local settings. Further, our findings indicate that the home-visit delivery channel correlates most strongly to high adherence among Yi caregivers, while the township-based channel influences high adherence among Han caregivers.

Despite delivery frequency’s showing no significant association with proper usage, higher frequency does indicate higher adherence to the proposed usage patterns of MNP across all three ethnic groups. When MNP is accessed more frequently, there is a greater probability that caregivers will adhere to usage instructions more regularly, which would thus increase adherence rates. This finding also has been observed in rural Nigeria, where higher frequency leads to increased adherence, which also may be due to increased frequency of communication between caregivers and distributors [42]. More frequent access to MNP implies a more regular supply and more contact with healthcare workers, which may act as an external motivation for caregivers to increase their adherence to MNP [56, 75]. Moreover, regular distribution is recommended by the National Institute of Nutrition and Health in China [74].

In summary, several practical recommendations can be drawn from the findings and conclusions of this study. First, a wider range of public information, such as pamphlets, brochures, and nutrition packaging and instructions, in more dialects and languages is needed to distribute MNP more effectively to caregivers in rural China. Second, townships doctors could be called upon to act as important actors in policy and interventions that aim to improve MNP proper usage among minority populations. Third, regular monthly MNP distribution is highly recommended for achieving high levels of MNP adherence, and may be an important focus for future research and/or policy initiatives.

### Strengths and limitations

This study has several strengths. First, this study explores the association between delivery patterns and the MNP feeding behaviors of caregivers and provides critical information on the role of delivery patterns in MNP usage. In this way, this study addresses a major gap in prior research. Second, our study sample includes caregivers from multiple ethnic minority groups that are often underrepresented in research. To this end, this study provides data that can be directly used by local health officers and administrators to improve the efficiency of MNP programs in China and other low-income countries.

This study also has several limitations. First, because our study presents the first observational findings on the associations between MNP delivery patterns and MNP feeding behaviors in rural China, the findings cannot be interpreted as indicating causality. Second, our study focuses on data solely from the perspective of caregivers, which provides evidence from only the demand side of MNP distribution. To better understand the differences between ethnicities and how access and use MNP and improve child health outcome, future research could include data from the supply side (through MNP distribution employees and programmers) to develop the most appropriate and effective delivery patterns for both the demand and supply sides.

## Conclusion

In conclusion, this study finds that caregivers from ethnic minority groups report lower levels of proper MNP usage than do Han caregivers, and Tibetan caregivers report the lowest rates of adherence to MNP. In addition, this study finds evidence of correlation between MNP delivery channel and both proper usage and high adherence as well as a correlation between MNP delivery frequency and high adherence. These preliminary data warrant more detailed and multi-ethnic investigations into the distribution of MNP in rural areas for future research and policy initiatives. The findings also can be used to inform policymakers about the access of minority groups to MNP as well as their levels of usage. Ultimately, we hope that the findings are able to motivate policymakers to explore the nature of MNP distribution as a way of addressing the Child Nutrition Improvement Implementation Programs in MNP distribution. Previous research indicates that children in rural areas of China continue to suffer from undernutrition, especially in ethnic minority areas. Despite free access to MNP, there remain barriers to proper usage and high adherence to MNP programs.

## Supplementary Information

**Additional File 1: Appendix Table 1.** The attrition analysis of households included and excluded in the final analytical sample. An attrition analysis of the 1021 caregivers who were included in the final analytical sample and the 215 caregivers who were not included in the final analytical sample. **Appendix Table 2.** Variables’ Description. Descriptions of the survey questions asked to survey participants. **Appendix Table 3.** Associations between MNP delivery patterns and feeding behaviors (unadjusted logistic regressions). Univariate analysis results of unadjusted logistic regressions to supplement **Table 3**. **Appendix Table 4.** Associations between MNP delivery patterns and MNP feeding behaviors. Overall results of the multivariate analysis (when the regression analysis controlled for demographic characteristics).

## Data Availability

The datasets generated during and/or analyzed during the current study are not publicly available due to institutional policy but are available from the corresponding author on reasonable request.

## Abbreviations

MNP: Micronutrient powder
OR: Odds Ratio
LMICs: Low- and middle-income countries
IDA: Iron-deficiency anemia
WHO: World Health Organization
SD: Standard deviation
AHI: Annual household incomes

## References

[1] WHO. Malnutrition. 2020. https://www.who.int/news-room/fact-sheets/detail/malnutrition. Accessed 13 Apr 2021.

[2] Black RE, Victora CG, Walker SP, Bhutta ZA, Christian P, de Onis M (2013). Maternal and child undernutrition and overweight in low-income and middle-income countries. Lancet..

[3] Luo R, Shi Y, Zhou H, Yue A, Zhang L, Sylvia S (2015). Micronutrient deficiencies and developmental delays among infants: evidence from a cross-sectional survey in rural China. BMJ Open.

[4] World Bank Group and UNICEF. Ending extreme poverty: a focus on children. https://www.unicef.org/reports. Accessed 16 Apr 2021.

[5] Lozoff B (2007). Iron deficiency and child development. Food Nutr Bull.

[6] UNICF. Vitamins & minerals for children fortifies economic development in China. UNICEF. https://www.unicef.org/media/media_23416.html. Accessed 16 Apr 2021.

[7] Victora CG, Adair L, Fall C, Hallal PC, Martorell R, Richter L (2008). Maternal and child undernutrition: consequences for adult health and human capital. Lancet..

[8] Local Burden of Disease Child Growth Failure Collaborators (2020). Mapping child growth failure across low- and middle-income countries. Nature..

[9] GBD (2018). 2017 mortality collaborators. Global, regional, and national age-sex-specific mortality and life expectancy, 1950-2017: a systematic analysis for the global burden of disease study 2017. Lancet..

[10] de Barros SF, Cardoso MA (2016). Adherence to and acceptability of home fortification with vitamins and minerals in children aged 6 to 23 months: a systematic review. BMC Public Health.

[11] Ritchie H, Roser M. Micronutrient Deficiency. Our World in Data. 2017.

[12] Thompson J, Biggs B-A, Pasricha S-R (2013). Effects of daily iron supplementation in 2- to 5-year-old children: systematic review and meta-analysis. Pediatrics..

[13] Stevens GA, Finucane MM, De-Regil LM, Paciorek CJ, Flaxman SR, Branca F (2013). Global, regional, and national trends in haemoglobin concentration and prevalence of total and severe anaemia in children and pregnant and non-pregnant women for 1995-2011: a systematic analysis of population-representative data. Lancet Glob Health.

[14] Stevens GA, Bennett JE, Hennocq Q, Lu Y, De-Regil LM, Rogers L (2015). Trends and mortality effects of vitamin a deficiency in children in 138 low-income and middle-income countries between 1991 and 2013: a pooled analysis of population-based surveys. Lancet Glob Health.

[15] Hess SY (2017). National Risk of zinc deficiency as estimated by National Surveys. Food Nutr Bull.

[16] Avula R, Frongillo EA, Arabi M, Sharma S, Schultink W (2011). Enhancements to nutrition program in Indian integrated child development services increased growth and energy intake of children. J Nutr.

[17] Sun J, Dai Y, Zhang S, Huang J, Yang Z, Huo J (2011). Implementation of a programme to market a complementary food supplement (Ying Yang Bao) and impacts on anaemia and feeding practices in Shanxi. China Matern Child Nutr.

[18] De-Regil LM, Suchdev PS, Vist GE, Walleser S, Peña-Rosas JP (2013). Home fortification of foods with multiple micronutrient powders for health and nutrition in children under two years of age (review). Evid-Based Child Health.

[19] Heidkamp RA (2017). Evidence for the effects of complementary feeding interventions on the growth of infants and young children in low- and middle-income countries. Nestle Nutr Inst Workshop Ser.

[20] Suchdev PS, Ruth LJ, Woodruff BA, Mbakaya C, Mandava U, Flores-Ayala R (2012). Selling sprinkles micronutrient powder reduces anemia, iron deficiency, and vitamin a deficiency in young children in Western Kenya: a cluster-randomized controlled trial. Am J Clin Nutr.

[21] Jefferds MED, Mirkovic KR, Subedi GR, Mebrahtu S, Dahal P, Perrine CG (2015). Predictors of micronutrient powder sachet coverage in Nepal. Matern Child Nutr.

[22] D’Agostino A, Ssebiryo F, Murphy H, Cristello A, Nakiwala R, Otim K, et al. Facility- and community-based delivery of micronutrient powders in Uganda: opening the black box of implementation using mixed methods. Matern Child Nutr. 2019;15. 10.1111/mcn.12798 PMC7199063 31622038

[23] Black RE, Allen LH, Bhutta ZA, Caulfield LE, de Onis M, Ezzati M (2008). Maternal and child undernutrition: global and regional exposures and health consequences. Lancet..

[24] Zou S, Liu Y, Zheng A, Huang Z (2021). Associations between dietary patterns and anaemia in 6- to 23-month-old infants in central South China. BMC Public Health.

[25] The World Bank. Prevalence of stunting, height for age (% of children under 5) Data. 2019. https://data.worldbank.org/indicator/sh.sta.stnt.zs. Accessed 10 May 2021.

[26] China 0–6 Years Old Children’s Nutrition Development Report (2012). 2015. http://www.chinanutri.cn/yyjkzxpt/yyjkkpzx/xcclk/xinxi/201501/t20150115_109818.html. Accessed 16 Apr 2021.

[27] Zhou J, Li D, Liu R (2020). Meta-analysis of the prevalence of iron deficiency anemia in infants and children aged 0 to 3 years in China from 2010 to 2019. China Matern Child Health.

[28] Li L-L, Wu Y-Y, Lei P-C, Sun C, Ye R-X, Wang Q-Z (2021). Study on the relationship between first-time complementary feeding practice and the nutritional status of infants and young children in the multi-ethnic rural areas of Sichuan Province. Sichuan Da Xue Xue Bao Yi Xue Ban.

[29] Li Z, Hu L (2015). Status survey and discussion of infant feeding at 0~23 months from 15 ethnic groups in rural area in Yunnan province. Chinese journal of child. Health care.

[30] WHO. Guideline. Use of multiple micronutrient powders for home fortification of foods consumed by infants and children 6–23 months of age. Geneva, world health. Organization. 2011. 24501787

[31] Luo R, Yue A, Zhou H, Shi Y, Zhang L, Martorell R (2017). The effect of a micronutrient powder home fortification program on anemia and cognitive outcomes among young children in rural China: a cluster randomized trial. BMC Public Health.

[32] Zhang WJ, Yang H, Peng M, Li P (2015). Analysis on the influencing factors of nutrition package administration of children nutrition improvement project in poor area of a county. Chinese J Women Children Health.

[33] Zhou X, Fang J, Luo J, Wang H (2017). Factors associated with taking Yingyangbao efficiently among infants and young children aged 6-24 months in poor rural areas of Hunan Province. China J Hygiene Res.

[34] Li F, Fang X, Liu J (2019). Analysis of the factors influencing the consumption of nutrition packages for children in poor areas in Gansu Province.

[35] Qu P-F, Zhang Y, Li J-M, Zhang R, Yang J-M, Lei F-L (2018). Complementary feeding patterns among ethnic groups in rural western China. J Zhejiang Univ Sci B.

[36] People’s Government of Sichuan Province. Overview of Sichuan. https://www.sc.gov.cn/10462/10758/11799/11800/2018/4/30/10300452.shtml. Accessed 28 Jul 2021.

[37] China Minzu Cultural Resources. Overview of Ethnic Regions 2017. http://minzunet.cn/eportal/ui?pageId=595416&articleKey=637166&columnId=614611. Accessed 8 Dec 2021.

[38] Ruoergai County government office. Overview of Ruoergei County 2021. http://www.ruoergai.gov.cn/regxrmzf/c100125/201908/2d4ee5b5d9bc4ec3a8a91b246ae9d02d.shtml?pageId=595416&articleKey=637166&columnId=614611. Accessed 8 Dec 2021.

[39] Kangding County government office. Overview of Kangding County 2021. http://www.kangding.gov.cn/kdsrmzf/c100437/zjby.shtml. Accessed 8 Dec 2021.

[40] Butuo County government office. Overview of Butuo County 2021. http://www.bt.gov.cn/mlbt/btgk/mjrk/201709/t20170905_346770.html. Accessed 8 Dec 2021.

[41] Meigu County government office. Overview of Meigu County 2021. http://www.meigu.gov.cn/nmlmg/mgkk/rkmz_40390/202105/t20210507_1901659.html. Accessed 8 Dec 2021.

[42] Guangyuan County Government Office. Overview of Guanyuan County. https://www.cngy.gov.cn/artic/show/20160720152014298.html. Accessed 9 Dec 2021.

[43] Yilong County government office. Overview of Yilong County 2021. http://www.yilong.gov.cn/invest/nation/index.html. Accessed 9 Dec 2021.

[44] Liu R, Ye R, Leng F, Sun C, Wang Q, Zhou H. High adherence and its influencing factors on multiple micronutrient powders (MNPs). Matern Child Nutr. 2021:e13278. 10.1111/mcn.13278 PMC8710102 34658128

[45] National Health Commission. The Technical Protocol of Children Nutrition Improvement Program in Poor Areas. http://www.gov.cn/xinwen/2019-09/05/content_5427467.htm. Accessed 11 Oct 2021.

[46] Madley-Dowd P, Hughes R, Tilling K, Heron J (2019). The proportion of missing data should not be used to guide decisions on multiple imputation. J Clin Epidemiol.

[47] Klebanoff MA, Cole SR (2008). Use of multiple imputation in the epidemiologic literature. Am J Epidemiol.

[48] Groves R, Cialdini R, Couper M. Understanding the decision to participate in a survey. Public Opinion Quarterly - PUBLIC OPIN QUART. 1992;56.

[49] Cheung KL, ten Klooster PM, Smit C, de Vries H, Pieterse ME (2017). The impact of non-response bias due to sampling in public health studies: a comparison of voluntary versus mandatory recruitment in a Dutch national survey on adolescent health. BMC Public Health.

[50] Wang Y, Cui C, Zhang Y, Wang L (2021). Factors associated with sleep quality among “left-behind women” in rural China: a cross-sectional study. Sleep Breath.

[51] Huang H, Liu S, Cui X, Zhang J, Wu H (2018). Factors associated with quality of life among married women in rural China: a cross-sectional study. Qual Life Res.

[52] Shi H, Zhang J, Du Y, Zhao C, Huang X, Wang X (2020). The association between parental migration and early childhood nutrition of left-behind children in rural China. BMC Public Health.

[53] Yi J, Zhong B, Yao S (2014). Health-related quality of life and influencing factors among rural left-behind wives in Liuyang. China BMC Womens Health.

[54] People’s government of Sichuan Province. Sichuan’s “migrant workers economy” Breaks the Trillion Mark. 2021. https://www.sc.gov.cn/10462/10464/10797/2021/1/26/26cecbe14e554073b0f0bc9473d7ff1a.shtml. Accessed 5 Jun 2022.

[55] Xie Y, Guo Q, Meng Y (2021). The health service use of aged rural-to-urban migrant workers in different types of cities in China. BMC Health Serv Res.

[56] Korenromp EL, Adeosun O, Adegoke F, Akerele A, Anger C, Ohajinwa C (2016). Micronutrient powder distribution through maternal, neonatal and child health weeks in Nigeria: process evaluation of feasibility and use. Public Health Nutr.

[57] Nguyen M, Poonawala A, Leyvraz M, Berger J, Schofield D, Nga TT (2016). A delivery model for home fortification of complementary foods with micronutrient powders: innovation in the context of Vietnamese health system strengthening. Nutrients..

[58] Fan S, Lin T (2017). Survey on Enshi Ganchang culture from the perspective of cultural anthropology. J Guangxi Sci Technol Teacher’s College.

[59] Mirkovic KR, Perrine CG, Subedi GR, Mebrahtu S, Dahal P, Staatz C (2016). Predictors of micronutrient powder intake adherence in a pilot programme in Nepal. Public Health Nutr.

[60] China government website. Chinese Language and Writing 2005. http://www.gov.cn/ziliao/zgjk/2005-06/16/content_6821.htm. Accessed 18 Dec 2021.

[61] Creed-Kanashiro H, Bartolini R, Abad M, Arevalo V (2016). Promoting multi-micronutrient powders (MNP) in Peru: acceptance by caregivers and role of health personnel. Matern Child Nutr.

[62] Kodish S, Rah JH, Kraemer K, de Pee S, Gittelsohn J (2011). Understanding low usage of micronutrient powder in the Kakuma refugee camp, Kenya: findings from a qualitative study. Food Nutr Bull.

[63] Brewer JD, Santos MP, Román K, Riley-Powell AR, Oberhelman RA, Paz-Soldan VA (2020). Micronutrient powder use in Arequipa, Peru: barriers and enablers across multiple levels. Matern Child Nutr.

[64] Samuel A, Brouwer ID, Pamungkas NP, Terra T, Lelisa A, Kebede A (2021). Determinants of adherence to micronutrient powder use among young children in Ethiopia. Matern Child Nutr.

[65] Kyei-Arthur F, Situma R, Aballo J, Mahama AB, Selenje L, Amoaful E (2020). Lessons learned from implementing the pilot micronutrient powder initiative in four districts in Ghana. BMC Nutrition.

[66] Roschnik N, Diarra H, Dicko Y, Diarra S, Stanley I, Moestue H (2019). Adherence and acceptability of community-based distribution of micronutrient powders in southern Mali. Maternal & Child Nutrition.

[67] Angdembe MR, Choudhury N, Haque MR, Ahmed T (2015). Adherence to multiple micronutrient powder among young children in rural Bangladesh: a cross-sectional study. BMC Public Health.

[68] Lundeen E, Schueth T, Toktobaev N, Zlotkin S, Hyder SMZ, Houser R (2010). Daily use of sprinkles micronutrient powder for 2 months reduces anemia among children 6 to 36 months of age in the Kyrgyz Republic: a cluster-randomized trial. Food Nutr Bull.

[69] Wu Q, Zhang Y, Chang S, Wang W, Helena van Velthoven M, Han H, et al. Monitoring and evaluating the adherence to a complementary food supplement (Ying Yang Bao) among young children in rural Qinghai, China: a mixed methods evaluation study. J Glob Health 2017;7:011101. 10.7189/jogh.07.011101 PMC5502707 28702176

[70] Niu H, Wang Y, Tang H, Gong L. Adherence and its influencing factors on the consumption of nutrition packs among poor rural children in Guizhou, Yunnan and Shanxi provinces. Journal of hygiene. Research. 2017; 10.19813/j.cnki.weishengyanjiu.2017.02.017.

[71] Giovannini M, Sala D, Usuelli M, Livio L, Francescato G, Braga M (2006). Double-blind, placebo-controlled trial comparing effects of supplementation with two different combinations of micronutrients delivered as sprinkles on growth, anemia, and iron deficiency in cambodian infants. J Pediatr Gastroenterol Nutr.

[72] Reerink I, Namaste SM, Poonawala A, Nyhus Dhillon C, Aburto N, Chaudhery D (2017). Experiences and lessons learned for delivery of micronutrient powders interventions. Matern Child Nutr.

[73] Ruixue Y. Health communication patterns and adherence to a micronutrient home fortification program among diverse ethnic groups in rural western China. J Nutr Educ Behav. 2021. 10.1016/j.jneb.2021.07.014 34690077

[74] National Health Commission, All-China Women's Federation (2014). Notice on the issuance of the program of nutrition improvement project for children in poor areas in.

[75] Health Behavior and Health Education | Part Two, Chapter Four : Integrated Behavior Model. https://www.med.upenn.edu/hbhe4/part2-ch4-integrated-behavior-model.shtml. Accessed 21 Oct 2021.

