# Supplementary material for: The association between micronutrient powder delivery patterns and caregiver feeding behaviors in rural China
Source: BMC Public Health. 2022 Jul 16;22:1366. doi: 10.1186/s12889-022-13726-4 (PMC9287899; doi:10.1186/s12889-022-13726-4)
Supplement: Supplementary file 1 — Additional File 1: Appendix Table 1. The attrition analysis of households included and excluded in the final analytical sample. An attrition analysis of the 1021 caregivers who were included in the final analytical sample and the 215 caregivers who were not included in the final analytical sample. Appendix Table 2. Variables’ Description. Descriptions of the survey questions asked to survey participants. Appendix Table 3. Associations between MNP delivery patterns and feeding behaviors (unadjusted logistic regressions). Univariate analysis results of unadjusted logistic regressions to supplement Table 3. Appendix Table 4. Associations between MNP delivery patterns and MNP feeding behaviors. Overall results of the multivariate analysis (when the regression analysis controlled for demographic characteristics). [file 12889_2022_13726_MOESM1_ESM.docx]

**Appendix Table 1. The attrition analysis of households included and excluded in the final analytical sample**

| **Variables** | **Included**  **(N = 1021)** | | **Data available** | **Excluded**  **(n =215)** | | ***P-value*** |
| --- | --- | --- | --- | --- | --- | --- |
|  | *N*/Mean | (*%*) / (SD) |  | *n* / Mean | (*%*) / (SD) |  |
| **Child characteristics** |  |  |  |  |  |  |
| Gender |  | | 208 |  | | *0.674* |
| Female | 491 | (48.1%) |  | 100 | (48.1%) |  |
| Male | 530 | (51.9%) |  | 108 | (51.9%) |  |
| Age (months) | 18.9 | (5.8) | 205 | 18.1 | (5.9) | *0.605* |
| Health status | 4.4 | (0.8) | 156 | 4.41 | (0.7) | *0.156* |
| **Caregiver characteristics** |  |  |  |  |  |  |
| Gender |  |  | 152 |  |  | *0.028* |
| Female | 912 | (89.3%) |  | 126 | (82.8%) |  |
| Male | 109 | (10.7%) |  | 26 | (17.2%) |  |
| Age (years) | 35.9 | (12.9) | 152 | 41.5 | (15.5) | *0.090* |
| Educational background |  |  | 150 |  |  | ***0.002*** |
| Never went to school | 476 | (46.6%) |  | 60 | (40.0%) |  |
| Did not complete elementary school | 117 | (11.5%) |  | 33 | (22.0%) |  |
| Completed elementary school | 132 | (12.9%) |  | 20 | (13.3%) |  |
| Completed primary school | 170 | (16.7%) |  | 28 | (18.6%) |  |
| Completed high school or above | 126 | (12.3%) |  | 9 | (6.1%) |  |
| Occupation |  |  | 148 |  |  | *0.203* |
| Farmers | 418 | (40.9%) |  | 52 | (35.1%) |  |
| Fulltime stay-at-home parents | 466 | (45.6%) |  | 81 | (54.7%) |  |
| Other | 137 | (13.5%) |  | 15 | (10.2%) |  |
| Annual household income (AHI) |  |  | 206 |  |  | *0.183* |
| < RMB 1.2k | 326 | (31.9%) |  | 54 | (26.2%) |  |
| ~ RMB 1.2k | 305 | (29.9%) |  | 57 | (27.6%) |  |
| ~ RMB 3.5k | 146 | (14.3%) |  | 35 | (16.9%) |  |
| ≥ RMB 5k | 244 | (23.9%) |  | 60 | (19.1%) |  |
|  |  |  |  |  |  |  |
| *Delivery Channel* ^a^ |  |  | 184 |  |  | *0.622* |
| Village-based | 291 | (28.5%) |  | 46 | (25.0%) |  |
| Township-based | 542 | (53.1%) |  | 102 | (55.4%) |  |
| Home-visit | 188 | (18.4%) |  | 36 | (19.6%) |  |
| *Delivery Frequency* |  |  | 186 |  |  | ***0.001*** |
| ≥ 3 months | 399 | (39.1%) |  | 45 | (24.1%) |  |
| 2 months | 113 | (11.1%) |  | 29 | (15.5%) |  |
| ≤ 1 month | 509 | (49.9%) |  | 112 | (60.2%) |  |
| *Proper usage* ^b^ |  |  |  |  |  | *0.566* |
| Yes | 755 | (74.0%) | 175 | 133 | (76.0%) |  |
| No | 266 | (26.1%) |  | 42 | (24.0%) |  |
| *Adherence* ^c^ |  |  | 38 |  |  |  |
| Low | 254 | (24.9%) |  | 15 | (39.4%) | *0.054* |
| High | 767 | (75.1%) |  | 23 | (60.6%) |  |

Source: authors’ survey. Notes: “MNP” refers to micronutrient powders. Data available refers to data collected but not included into final analysis due to varied reasons (See details in Methods description). Child age, child health status, and caregiver age are all listed in mean (SD); all other variables listed in frequency (percentage). Child health status was reported by caregivers on a scale of 1 to 5: 1 = very poor; 2 = poor; 3 = fair; 4 = good; or 5 = very good.

^a^ Village-based delivery channel: MNP were distributed at village health office, village activity room or village committee location; township-based: MNP were distributed at township health center; home-visit delivery: MNP were distributed to their household by home-visit.

**Appendix Table 2. Variables’ Description**

Notes: MNP refers to micronutrient powders.

^a^ “other” occupations include self-employed individuals, drivers, for example.

| Variables | Items | Survey Questions and Measures |
| --- | --- | --- |
| ***Feeding behavior*** | a. Proper usage of MNP | *What methods do you usually use to feed your child MNP?*  1 = adding MNP to warm boiling water and stirring into a paste  2 = mixing MNP with other supplementary food  3 = other. |
|  | b. Adherence to MNP | *How many sachets of MNP do you feed your child every week?*  1= ≥ 4 sachets/week (high adherence)  0 = < 4 sachets/week (low adherence) |
| ***Delivery patterns*** | a. Delivery Channel | *What kind of delivery channel do you currently access MNP primarily?*  1 = village-based (MNP were distributed at the village health office, village activity room or village committee location)  2 = township-based (MNP were distributed at township health center)  3 = home-visit (MNP were distributed to their household by home-visit) |
|  | b. Delivery frequency | *How frequently do you currently access MNP on average?*  1= ≥ 3 months (access MNP more than every 3 months)  2 = 2 months (access MNP every 2 months)  3= ≤ 1 month (access MNP every month or multiple times per month) |
| ***Control variables*** | 1. Child gender; age | *Is your child male or female?*  *How old is you child, in months?* |
|  | 1. Child health status | *How is your child’s health now?*  1 = very poor  2 = poor  3 = fair  4 = good  5 = very good |
|  | c. Caregiver Demographic information: educational background, ethnicity; occupation | *What is your highest level of education?*  1 = never went to school  2 = did not complete elementary school  3 = completed elementary school  4 = completed primary school  5 = completed high school or above  *What is your ethnicity?*  1 = Yi  2 = Tibetan  3 = Han  *What is your main occupation now?*  1 = farmer  2 = fulltime stay-at-home parent  3 = other^a^ |
|  | d. Annual household income | *What is your annual household income?*  1 = < RMB 1.2k  2 = ~ RMB 1.2k  3 = ~ RMB 3.5k  4 = ≥ RMB 5k |

**Appendix Table 3 Associations between MNP delivery patterns and feeding behaviors (unadjusted logistic regressions)**

|  | **Proper usage ^b^ (OR)** | | | |  | **Adherence ^c^ (OR)** | | | |
| --- | --- | --- | --- | --- | --- | --- | --- | --- | --- |
| **Variables** | Total | Han | Tibetan | Yi |  | Total | Han | Tibetan | Yi |
| **Channel ^a^** (Village-based as reference) | | | | | | | | | |
| Township-based | **3.3***** | **2.1*** | **2.7***** | **1.8*** |  | **1.3*** | **2.4**** | **2.3**** | 1.3 |
|  | (0.6) | (0.9) | (0.9) | (0.6) |  | (0.2) | (0.8) | (0.7) | (0.5) |
| Home-visit | 1.1 | 1.0 | 1.8 | 1.0 |  | **2.5***** | N/A | **4.6***** | 1.7 |
|  | (0.2) | (0.8) | (0.7) | (0.3) |  | (0.6) | N/A | (2.1) | (0.5) |
| **Frequency** (≥ 3 months as reference) | | | | | | | | | |
| 2 months | 0.9 | 1.1 | 1.3 | 0.5 |  | **1.9***** | **2.4**** | **3.1**** | 1.2 |
|  | (0.2) | (0.5) | (0.6) | (0.2) |  | (0.5) | (1.0) | (1.5) | (0.6) |
| ≤ 1 months | 1.0 | 1.6 | 0.9 | 0.8 |  | **3.3***** | **3.1***** | **4.7***** | **4.3***** |
|  | (0.2) | (0.6) | (0.3) | (0.2) |  | (0.6) | (0.9) | (1.4) | (1.4) |

Source: authors’ survey. Notes: MNP refers to micronutrient powders; odds ratios were reported; standard errors in parentheses; the results of control variables were not included.

*p < .05. **p < .01. ***p < .001.

^a^ Village-based delivery channel: MNP were distributed at village health office, village activity room or village committee location; Township-based delivery channel: MNP were distributed at township health center; Home-visit delivery channel: MNP were distributed to their household by home-visit.

^b^ Proper usage (Yes) refers to following the MNP feeding instructions: Adding MNP to warm boiling water and stir into paste, and mixing MNP with other supplementary food, No refers to other usage

^c^ High adherence: ≥4 MNP sachets was consumed every week, low adherence: <4 MNP sachets was consumed every week.

**Appendix Table 4. Associations between MNP delivery patterns and feeding behaviors**

| **Variables** | **Proper usage ^b^ (OR)** | | | |  | **Adherence ^c^ (OR)** | | | |
| --- | --- | --- | --- | --- | --- | --- | --- | --- | --- |
|  | Total | Han | Tibetan | Yi |  | Total | Han | Tibetan | Yi |
| ***MNP delivery channel* ^a^** (Village-based as ref.) | | | | | | | | | |
| Township-based | **2.6***** | 2.1 | **3.5***** | **2.0**** |  | **1.7***** | **2.2**** | **2.6***** | 1.4 |
|  | (0.5) | (0.9) | (1.2) | (0.6) |  | (0.3) | (0.8) | (0.9) | (0.5) |
| Home-visit | 1.1 | 0.9 | 1.8 | 0.9 |  | **2.3***** | N/A | **4.8***** | 1.5 |
|  | (0.2) | (0.7) | (0.8) | (0.3) |  | (0.6) | N/A | (2.4) | (0.5) |
| ***MNP feeding frequency*** (≥ 3 months as ref.) | | | | | | | | | |
| 2 months | 0.8 | 1.1 | 1.8 | 0.5 |  | **2.2***** | **2.6**** | **3.9***** | 1.2 |
|  | (0.2) | (0.5) | (0.9) | (0.2) |  | (0.6) | (1.1) | (1.9) | (0.6) |
| ≤ 1 month | 0.9 | 1.6 | 1.0 | 0.8 |  | **3.5***** | **3.1***** | **5.8***** | **4.3***** |
|  | (0.2) | (0.6) | (0.3) | (0.2) |  | (0.6) | (0.9) | (1.8) | (1.5) |
| ***Control variables*** |  |  |  |  |  |  |  |  |  |
| Child gender (1 = male) | 0.9 | 1.0 | 0.7 | 1.0 |  | 0.9 | (0.3) | (0.2) | (0.3) |
|  | (0.1) | (0.4) | (0.2) | (0.2) |  | (0.1) | 1.0 | 0.9 | 0.9 |
| Child age (months) | 0.9 | 1.0 | 0.9 | 0.9 |  | 1.0 | (0.03) | (0.02) | (0.02) |
|  | (0.01) | (0.03) | (0.02) | (0.02) |  | (0.01) | 0.8 | 1.0 | 0.8 |
| Child Health **^d^** | **0.8**** | 0.8 | **0.6**** | 1.1 |  | **0.8*** | (0.2) | (0.2) | (0.2) |
|  | (0.1) | (0.2) | (0.1) | (0.2) |  | (0.1) | 2.2** | 2.6*** | 1.4 |
| Caregiver gender (1 = male) | 0.7 | 0.7 | 0.6 | 1.4 |  | 0.9 | (0.5) | (0.7) | (0.3) |
|  | (0.2) | (0.4) | (0.2) | (0.6) |  | (0.2) | 1.02 | 1.0 | **1.0**** |
| Caregiver age (years) | **1.0***** | 1.0 | 1.0 | **1.0**** |  | **1.0***** | (0.02) | (0.01) | (0.0) |
|  | (0.01) | (0.01) | (0.01) | (0.01) |  | (0.01) | 0.9 | **0.8**** | 1.1 |
| Caregiver educational level **^e^** | **1.2***** | 0.9 | 1.0 | 1.2 |  | 0.9 | (0.2) | (0.1) | (0.2) |
|  | (0.1) | (0.2) | (0.1) | (0.2) |  | (0.1) | 0.9 | 0.6 | 0.6 |
| Caregiver occupation **^f^** (1= Farmer) |  |  |  |  |  |  |  |  |  |
| Fulltime stay-at-home parent | 1.0 | 1.1 | 0.9 | 0.7 |  | **0.6**** | (0.5) | (0.2) | (0.3) |
|  | (0.2) | (0.6) | (0.3) | (0.2) |  | (0.1) | 0.8 | 0.8 | 0.5 |
| Others | 1.0 | 0.6 | **2.8**** | **0.3**** |  | **0.5***** | (0.5) | (0.3) | (0.3) |
|  | (0.3) | (0.4) | (1.3) | (0.2) |  | (0.1) | 0.9 | 0.9 | **0.6**** |
| Annual household income **^g^** | 1.1 | 1.0 | 1.2 | 0.8 |  | **0.8**** | (0.1) | (0.1) | (0.1) |
|  | (0.1) | (0.2) | (0.2) | (0.1) |  | (0.0) | 1.2 | 0.6 | 0.9 |

Source: authors’ survey. Notes: MNP refers to micronutrient powders; odds ratios were reported; standard errors in parentheses.; the results of control variables were not included. *p < .05. **p < .01. ***p < .001.

^a^ Village-based delivery channel: MNP were distributed at village health office, village activity room or village committee location; township-based: MNP were distributed at township health center; home-visit delivery: MNP were distributed to their household by home-visit.

^b^ Proper usage (yes) refers to following the MNP feeding instructions: Adding MNP to warm boiling water and stir into paste, and mixing MNP with other supplementary food; no refers to other usage.

^c^ High adherence: ≥4 MNP sachets was consumed every week, low adherence: <4 MNP sachets was consumed every week.

^d^ Child health status: 1 = very poor; 2 = poor; 3 = fair; 4 = good; or 5 = very good.

^e^ Caregiver educational level: 1 = never attended school; 2 = did not complete elementary school; 3 = completed elementary school; 4 = completed primary school; or 5 = completed high school or above.

^f^ Caregiver occupation:1 = farmer; 2 = fulltime stay-at-home parent; or 3 = other (individual self-employed and drivers, etc.).

^g^ Annual household income 1 = < RMB 1.2k; 2 = ~ RMB 1.2k; 3 = ~ RMB 3.5k; or 4 = ≥ RMB 5k
